# Global hotspots and trends in tea anti-obesity research: a bibliometric analysis from 2004 to 2024

**DOI:** 10.3389/fnut.2024.1496582

**Published:** 2024-11-13

**Authors:** Shan Liu, Boyan Fan, Xiaoping Li, Guixiang Sun

**Affiliations:** ^1^College of Traditional Chinese Medicine, Hunan University of Chinese Medicine, Changsha, China; ^2^The Center for Treatment of Pre-disease, The First Affiliated Hospital of Hunan University of Chinese Medicine, Changsha, China

**Keywords:** obesity, bibliometric analysis, tea, epigallocatechin gallate, tea polyphenols, tea polysaccharides, gut microbiota, lipid metabolism

## Abstract

**Background:**

The prevalence of obesity and its related ailments is on the rise, posing a substantial challenge to public health. Tea, widely enjoyed for its flavors, has shown notable potential in mitigating obesity. Yet, there remains a lack of exhaustive bibliometric studies in this domain.

**Methods:**

We retrieved and analyzed multidimensional data concerning tea and obesity studies from January 2004 to June 2024, using the Web of Science Core Collection database. This bibliometric investigation utilized tools such as Bibliometrix, CiteSpace, and VOSviewer to gather and analyze data concerning geographical distribution, leading institutions, prolific authors, impactful journals, citation patterns, and prevalent keywords.

**Results:**

There has been a significant surge in publications relevant to this field within the last two decades. Notably, China, Hunan Agricultural University, and the journal Food and Function have emerged as leading contributors in terms of country, institution, and publication medium, respectively. Zhonghua Liu of Hunan Agricultural University has the distinction of most publications, whereas Joshua D. Lambert of The State University of New Jersey is the most cited author. Analyses of co-citations and frequently used keywords have identified critical focus areas within tea anti-obesity research. Current studies are primarily aimed at understanding the roles of tea components in regulating gut microbiota, boosting fat oxidation, and increasing metabolic rate. The research trajectory has progressed from preliminary mechanism studies and clinical trials to more sophisticated investigations into the mechanisms, particularly focusing on tea’s regulatory effects on gut microbiota.

**Conclusion:**

This study offers an intricate overview of the prevailing conditions, principal focus areas, and developmental trends in the research of tea’s role against obesity. It delivers a comprehensive summary and discourse on the recent progress in this field, emphasizing the study’s core findings and pivotal insights. Highlighting tea’s efficacy in obesity prevention and treatment, this study also points out the critical need for continued research in this area.

## 1 Introduction

Obesity, defined by an abnormal and excessive accumulation of fat, is witnessing a notable increase in its prevalence worldwide (1, 2). The World Health Organization (WHO) projects that by 2022, approximately 2.5 billion adults aged 18 and older will be classified as overweight globally, representing around 30% of the total population. Of these, around 890 million individuals are expected to be classified as obese (3, 4). The main objective of managing obesity is to enhance health, as achieving a sustained weight loss exceeding 10% of total body weight significantly improves various obesity-related conditions, including type 2 diabetes (T2D), hypertension, fatty liver disease, and obstructive sleep apnea, ultimately leading to a better quality of life (5).

Currently, anti-obesity medications predominantly focus on regulating food intake by acting on various neurotransmitters in the central nervous system (CNS). These medications are designed to reduce hunger, increase feelings of fullness, and diminish food reward, either individually or in combination (5). Beyond Orlistat, which inhibits gastric and pancreatic lipase activities resulting in the non-absorption of approximately 30–35% of ingested fats (6), other drugs such as Phentermine and Bupropion influence distinct neurological pathways (7, 8). The primary side effects of Orlistat are gastrointestinal, while Phentermine (with or without Topiramate) and Naltrexone-Bupropion are more prone to cause neuropsychiatric symptoms, such as agitation and insomnia (9, 10). Despite their market approval and availability, these drugs continue to pose safety concerns and are generally expensive (11–13). Additionally, while weight loss surgeries like adjustable gastric banding, Roux-en-Y gastric bypass, and sleeve gastrectomy are regarded as the most effective and long-lasting methods for managing obesity (14), only 1% of eligible patients undergo these procedures. The predominant barriers to surgery are its limited accessibility, substantial cost, patient hesitance, and the risk of severe complications and mortality, although the rates of surgery-related deaths have markedly decreased, the incidence of complications persists at about 17% (14).

Lifestyle adjustments, including dietary and physical activity changes, are fundamental to obesity management. Although dependent on personal commitment, they significantly reduce the risk of diseases associated with obesity, particularly in genetically predisposed individuals (15, 16). Other therapeutic options, such as pharmacological treatments and surgical interventions, have their own limitations, including potential adverse effects, and elevated risks and expenses (17, 18). Amid these challenges, the focus on natural foods as potential alternatives has increased, with tea being a notable example.

Tea, a beverage brewed from the leaves of the tea plant (*Camellia sinensis*), has been used in Chinese medicine for over 3,000 years, and it is an infusion made from the leaves of the tea plant (19, 20). Over centuries, tea has spread from China to other parts of Asia, Europe, the Americas, and Africa (19). Tea is classified based on its processing techniques into six main varieties: green tea (unfermented), white tea (lightly fermented), yellow tea (mildly fermented), oolong tea (semi-fermented), black tea (fully fermented), and dark tea (post-fermented) (21–23).

Green tea, the earliest form of tea, undergoes withering, fixation, rolling, and drying processes, with fixation crucial for deactivating enzymes at high temperatures, thereby preserving high levels of catechins and eliminating moisture and grassy smells from the leaves (24). This results in its distinctive “clear soup and green leaves” appearance and a rich, astringent taste. Green tea, minimally processed, retains more natural substances and undergoes less vitamin degradation (25). Depending on the processing method, green tea is further classified into pan-fired, oven-baked, steamed, and sun-dried varieties. White tea, primarily produced in Fujian and Yunnan provinces of China, is celebrated for its unique flavor (26). Its processing involves only withering and drying, significantly reducing the moisture content of the leaves, enhancing enzyme activity, partially oxidizing polyphenols, and degrading chlorophyll, which contributes to its distinctive color, aroma, and flavor (27–29). White tea is particularly rich in catechins and their derivatives, especially epigallocatechin gallate (EGCG) (30).Yellow tea, unique to China, involves fixation, rolling, “yellowing,” and drying. The “yellowing” process, conducted in a humid and warm setting, allows for the transformation of metabolites in the tea leaves through the combined action of heat and enzymes, giving it the characteristic “yellow leaves and yellow soup” appearance and a sweeter, mellower taste than green tea (31). Yellow tea is also rich in phenolic compounds, amino acids, soluble sugars, vitamins, and other nutrients (32). Oolong tea, popular in southern China and classified as semi-fermented, combines the aromas of green tea with the rich flavors of black tea (33, 34). Its production includes several steps: sun withering, oxidation, tossing, rolling, firing, final roasting, and packaging (35). The partial oxidation of catechins during the fermentation process, ranging from 10 to 70%, reduces the catechin content but increases the concentration of polymerized polyphenols (36). Black tea, the most extensively consumed globally and accounting for the majority of tea production (37), is known for its health benefits, including anti-inflammatory, antidiabetic, antihypertensive, anticancer, and anti-obesity properties (38). Its processing involves withering, rolling, fermentation, and drying, during which tea polyphenols (TPs) undergo oxidation and polymerization to form compounds like theaflavins, thearubigins, and theabrownins, contributing to its distinctive color, flavor, and aroma (39). Pu-erh tea, originating from Yunnan province in China, is a post-fermented type noted for its unique aroma and flavor as well as various health benefits (40). Its primary processing step, “wo dui,” involves microbial fermentation through extracellular enzymes and biological heat, along with the metabolic processes of the tea leaves, leading to its distinctive flavor profile (41).

In food science and nutrition research, the health benefits of tea, particularly its potential to mitigate obesity and related diseases, have garnered increasing attention. Recent studies, including one utilizing a nonlinear dose–response assessment, have identified an inverse correlation between body weight, body mass index (BMI), and high levels of green tea consumption, specifically over 1,000 mg/day. Supplementation with green tea has been shown to reduce body weight and BMI in overweight and obese women, suggesting that healthcare professionals might recommend a daily intake of at least 1,000 mg of green tea for a minimum duration of 8 weeks (42).

Tea is abundant in active dietary compounds, and its regular consumption is associated with various health benefits, particularly in managing human metabolic diseases (43). Epidemiological studies have consistently demonstrated that regular consumption of tea and its components positively affects these diseases, reducing the risk of cardiovascular diseases, stroke, diabetes, metabolic syndrome, and obesity (44, 45). Experimental research has further investigated tea’s antioxidant, anti-inflammatory, anticancer, anti-obesity, cardiovascular protective, liver protective, and hypoglycemic activities and its underlying mechanisms (46). A meta-analysis has verified that supplements containing green tea catechins effectively reduce waist circumference and triglyceride levels while improving HDL-C levels (47). Additionally, black tea has been shown to alter the mRNA levels of liver lipid metabolism genes, thereby preventing excessive liver fat accumulation (48). By maintaining a balance between gut microbiota and liver lipids, supplementation with Huangshan Maofeng green tea extract (HTE) has significantly reduced fat accumulation in rats, improving conditions related to hyperlipidemia and hepatic steatosis (49). TPs have demonstrated significant (*p* < 0.01) effects in reducing the expression levels of COX-2 and iNOS, thereby decreasing liver fat content and degeneration. These findings suggest that TPs, through the involvement of TNF-α, IL-1 beta, and IL-6, could play a therapeutic role in treating obesity, liver inflammation, and fatty degeneration by inhibiting COX-2 and iNOS (50). Catechins have also been shown to prevent obesity-induced kidney damage by regulating the PPARγ/CD36 pathway and the rat intestinal-kidney axis (51).

Tea exhibits significant anti-obesity effects; however, the development of obesity is influenced by various factors, such as diet and exercise. Therefore, combining tea with other functional substances or adopting healthy lifestyles can more effectively promote weight loss and enhance weight management. Additionally, the stability of some substances is poor, and the application of nanotechnology and other methods can augment their strength and efficacy.

For instance, Winter Melon Lotus Leaf Tibetan Tea (WLTT) effectively mitigates obesity by modulating gut microbiota imbalances and stabilizing gut flora; its use is also considered safe. This blend of Tibetan tea with both medicinal and edible Chinese herbs presents a novel approach to combating obesity (52). Additionally, Compound Citrus Peel Tea (CCT), which is formulated from citrus peel, Ganoderma lucidum, and Pu-erh tea, manages gut microbiota and counters metabolic disorders related to obesity in mice (53). Research indicates that when green tea is augmented with *α*-glucosyl hesperidin (GT-GH), it prevents weight gain, especially in individuals younger than 50, where its anti-obesity effects are more pronounced (54). Moreover, a combination of Pu-erh tea extract and intermittent fasting (IF) addresses obesity by curbing fat accumulation and enhancing thermogenesis, which may reduce follicle-stimulating hormone (FSH) levels and thus alleviate FSH’s suppressive impact on UCP1 (55). When used alongside physical exercise, Green Tea Extract (GTE) not only improves metrics such as weight, BMI, waist-to-hip ratio, and body fat percentage, but it also significantly enhances anti-inflammatory and metabolic responses (56). Therefore, tea, as a viable natural remedy for obesity, effectively supports a healthy lifestyle.

EGCG, a natural polyphenolic compound, exhibits multiple biological activities, including antioxidant, antibacterial, anti-obesity, anti-inflammatory, and anticancer properties (57). Despite these properties, its clinical utility is restricted by its low stability under neutral or alkaline conditions and poor oral bioavailability. Techniques such as esterification, the use of nanoparticle technology, silicon-based EGCG nanoparticles (EGCG-NPs), and other EGCG formulations provide optimal strategies for its modification. These adaptations result in low-toxicity, high-concentration EGCG, which facilitates effective penetration and absorption both *in vitro* and *in vivo*. Enhancements to EGCG through reduced dosages markedly increase its biological activity, efficacy, and stability, thus expanding its use across clinical settings, and the food and cosmetics sectors (58). Encapsulating EGCG with nanotechnology can improve its stability, efficacy, and pharmacokinetic characteristics (59). Additionally, researchers have developed and characterized two novel EGCG-glucose conjugates: glu-EGCG, with one glucose molecule, and 2glu-EGCG, with two glucose molecules. Experimental outcomes on animals reveal that both glu-EGCG and 2glu-EGCG exhibit substantially greater antioxidant activities than EGCG alone, effectively diminishing ischemic regions, preventing morphological alterations in brain tissue, reducing neuronal loss, and balancing oxidizing and antioxidizing agents. Notably, 2glu-EGCG displays stronger antioxidant capabilities than glu-EGCG, highlighting their substantial potential as targeted antioxidant neuroprotective agents (60). Moreover, the combination of EGCG and L-theanine in green tea not only curbs fat accumulation but also, through forming a complex with L-theanine/*β*-cyclodextrin, significantly boosts the bioavailability of EGCG as well as its lipid-lowering and weight reduction impacts (61). Tea mixtures treated with nanotechnology might enhance their bioavailability and efficacy, showing robust anti-obesity properties (62). Through diverse methods like esterification and nanoparticle technology, the stability and biological activity of EGCG can be significantly improved, paving the way for its broader application in functional foods and beyond (58).

Bibliometrics, the quantitative evaluation of scientific literature via mathematical and statistical techniques, can uncover developmental trends, central themes, and emergent hotspots within specific disciplines (63). In recent years, various bibliometric tools have been extensively adopted across diverse research areas. Among these, VOSviewer, developed by Van Eck and his colleagues at Leiden University, Netherlands (64); crafted by ChanSuperMax at Drexel University, USA (65); and Bibliometrix, an R-based tool from Dr. Massimo Aria at the Università degli Studi Federico II in Naples, Italy (66), stand out. Each application has unique capabilities: VOSviewer is adept at illustrating keyword relationships and their associative strengths; CiteSpace excels at detecting developmental laws, pinpointing research hotspots, and defining field boundaries; Bibliometrix offers detailed graphical representations of data from literature (64, 67, 68).

Despite increasing studies into tea’s anti-obesity effects, supported by multiple animal and human research (69, 70), there is a noticeable dearth of extensive bibliometric studies in this realm, especially concerning the prediction of research focal points. This study utilizes Bibliometrix, CiteSpace, and VOSviewer for a bibliometric review of publications related to tea and obesity spanning the last two decades. The objective is to chart the current research landscape, delineate key hotspots, and project future trends, thereby establishing a basis for further inquiry.

## 2 Materials and methods

### 2.1 Data sources and methods

#### 2.1.1 Retrieval strategy

To ensure thorough and precise data collection, searches were performed within the Science Citation Index Expanded (SCI-E) and the Social Sciences Citation Index (SSCI) from the Web of Science (WOS) Core Collection. The deployed search strategy was: (TS = (tea) AND TS = (obesity OR “high-fat diet” OR “overweight” OR “obes*” OR “body mass index” OR BMI OR “adiposity” OR “excess weight” OR “weight gain”)), covering the period from January 1, 2004, to June 30, 2024. This search was executed on July 1, 2024, restricting the inclusion to articles and reviews.

#### 2.1.2 Data processing

**Figure 1:**
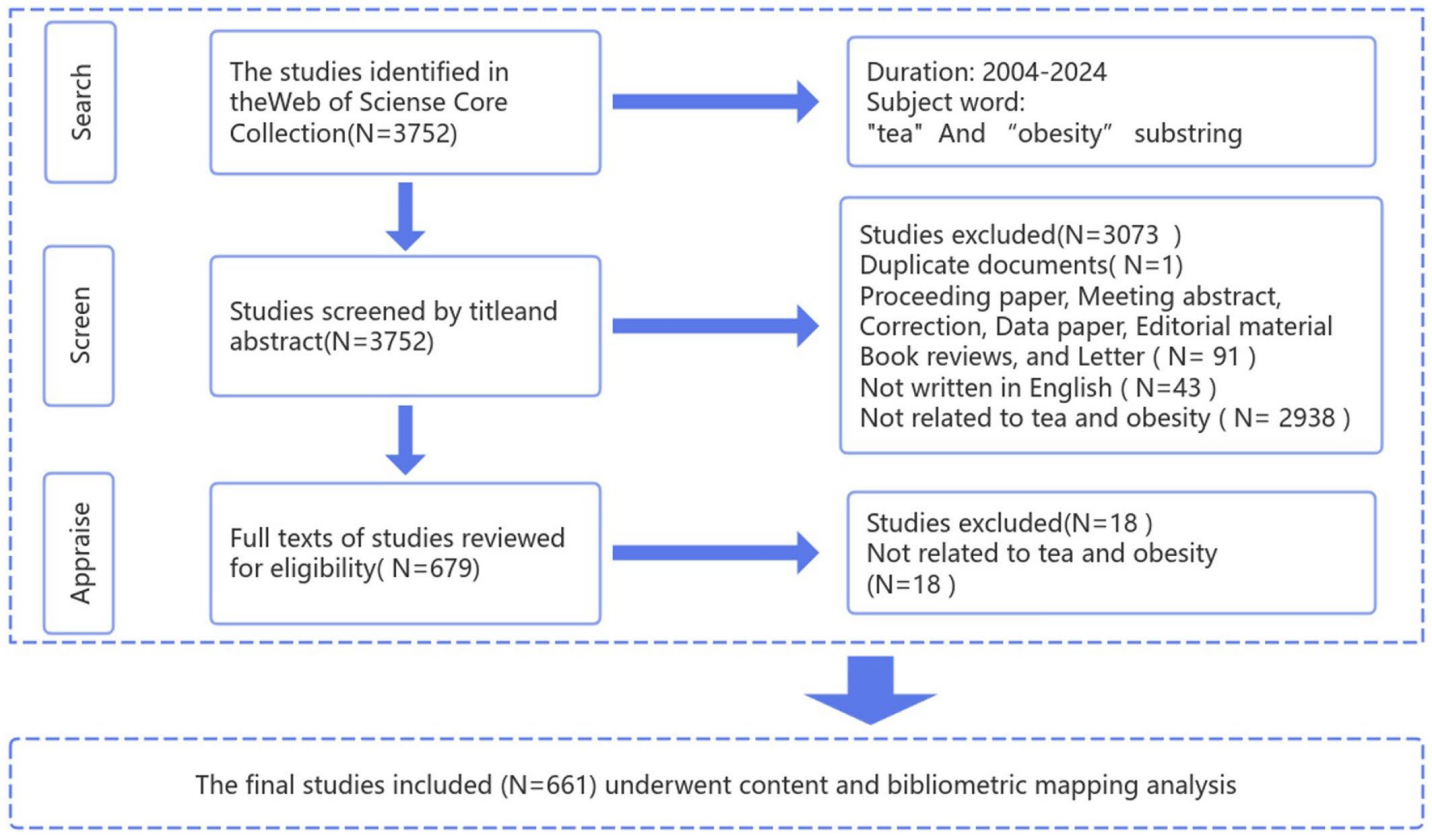
Screening flow chart.

To reduce biases from automated searches, a manual screening method was implemented, ensuring that only literature directly relevant to the anti-obesity properties of tea was analyzed for accurate and dependable assessment. Two researchers independently performed the literature search and download process. The initial search identified 3,752 potentially relevant articles, with 661 eventually selected for inclusion. Figure 1 illustrates the detailed retrieval and study selection methodology. Subsequently, after data validation and standardization, the literature was exported in both “Bibtex” and “plain text files,” which included comprehensive documents and cited references. These were analyzed further using VOSviewer (version 1.6.20), CiteSpace (version 6.2.R6), and the “bibliometrix” software package.

### 2.2 Bibliometric analysis

The bibliometric analysis focused on critical indicators such as titles, abstracts, keywords, authors, countries of publication, publication years, and journal details. For pivotal studies, the Web of Science (WOS) provided the most recent impact factors (IF), 5-year impact factors, journal tiers, and Hirsch index (H-index). The IF is an essential measure of a journal’s influence, determined by the citation frequency of its articles in subsequent scientific works (71). These indicators are vital for evaluating the quality of publications and serve as a core component of scholarly assessment (72). CiteSpace conducted a burst analysis of keywords and references for specific periods, aiding in the identification and clarification of emerging trends. Meanwhile, the “bibliometrix” package offered comprehensive statistics on journals, authors, countries, and institutions, while VOSviewer mapped collaborative networks among countries and institutions and conducted co-citation analyses of highly cited documents (73, 74).

## 3 Results

Utilizing the R package Bibliometrix, we conducted an analysis of the literature on tea anti-obesity research (Supplementary Figure S1). Over the period from 2004 to 2024, the field demonstrated a positive trajectory with a total of 661 publications, including 541 research papers and 120 review articles. These publications spanned 208 different journals, demonstrating the breadth of research interest in this area. On average, each publication received 46.1 citations, emphasizing the substantial impact of these studies. The mean age of these publications was 7.24 years, indicating a mature and well-established research continuum.

### 3.1 Annual trends in the number of publications

**Figure 2:**
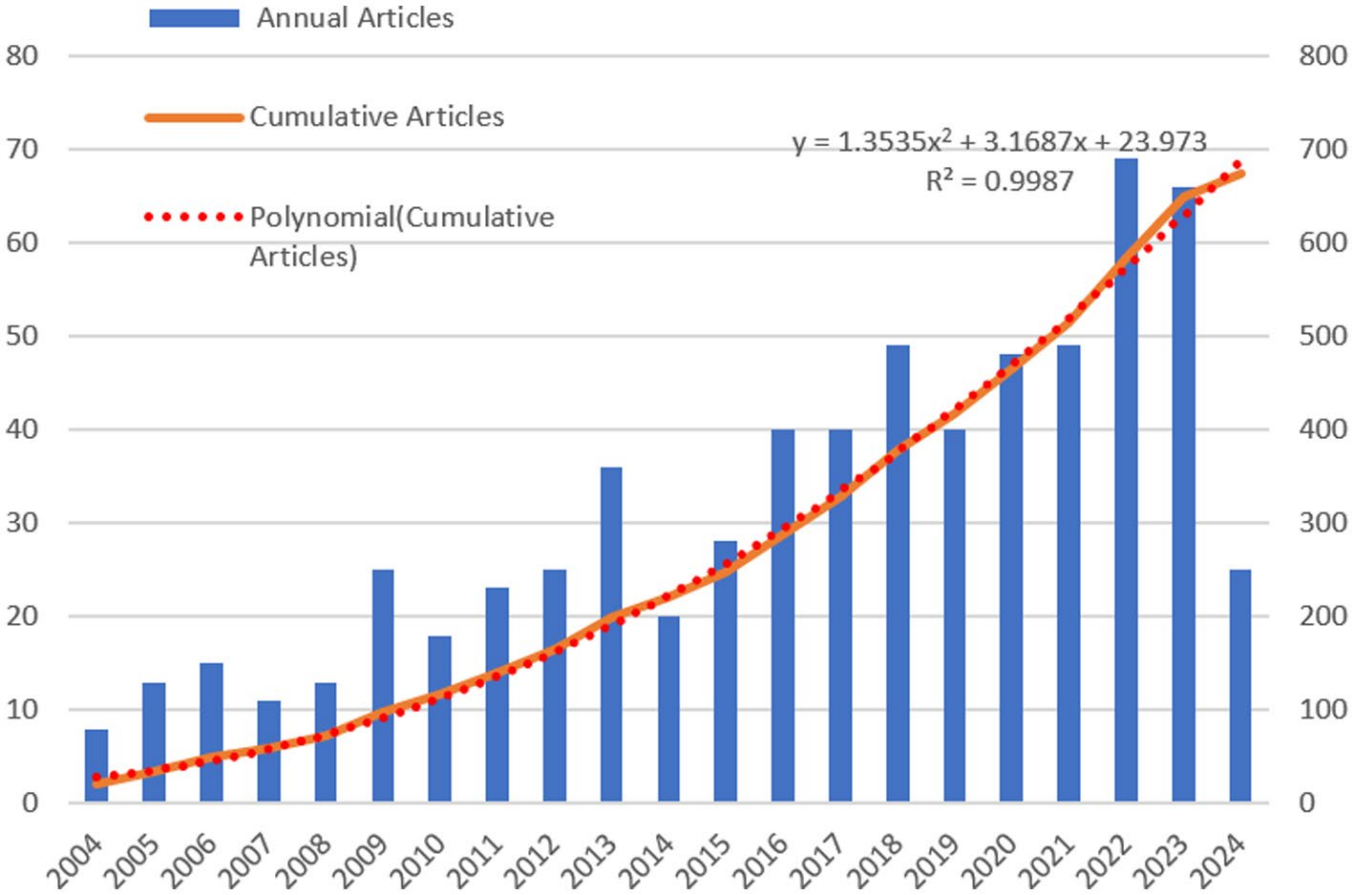
Publication growth trend of tea and obesity from 2004 to 2024. This graph shows annual publications in blue bars, the cumulative publications in an orange curve, and the red dashed line models the growth trend.

Figure 2 displays both the annual and cumulative publication trends from 2004 to 2024, illustrating the dynamic evolution of research within this domain. The data show a consistent annual increase in publications, with a marked rise beginning in 2016. Despite some variability in yearly publication numbers, the overall volume has grown steadily, with an average annual growth rate of 5.86%, pointing to an increasing focus on tea anti-obesity research. We employed a polynomial model y = 1.3535x^2^ + 3.1687x + 23.973 to describe the growth trend of cumulative publications, achieving a coefficient of determination (R^2^) of 0.9987. This result confirms that the polynomial model accurately represents the growth trend in the cumulative number of articles and predicts a continued increase, suggesting that tea anti-obesity research is an emerging and vibrant area of study.

### 3.2 Countries and affiliations collaborative network

**Figure 3:**
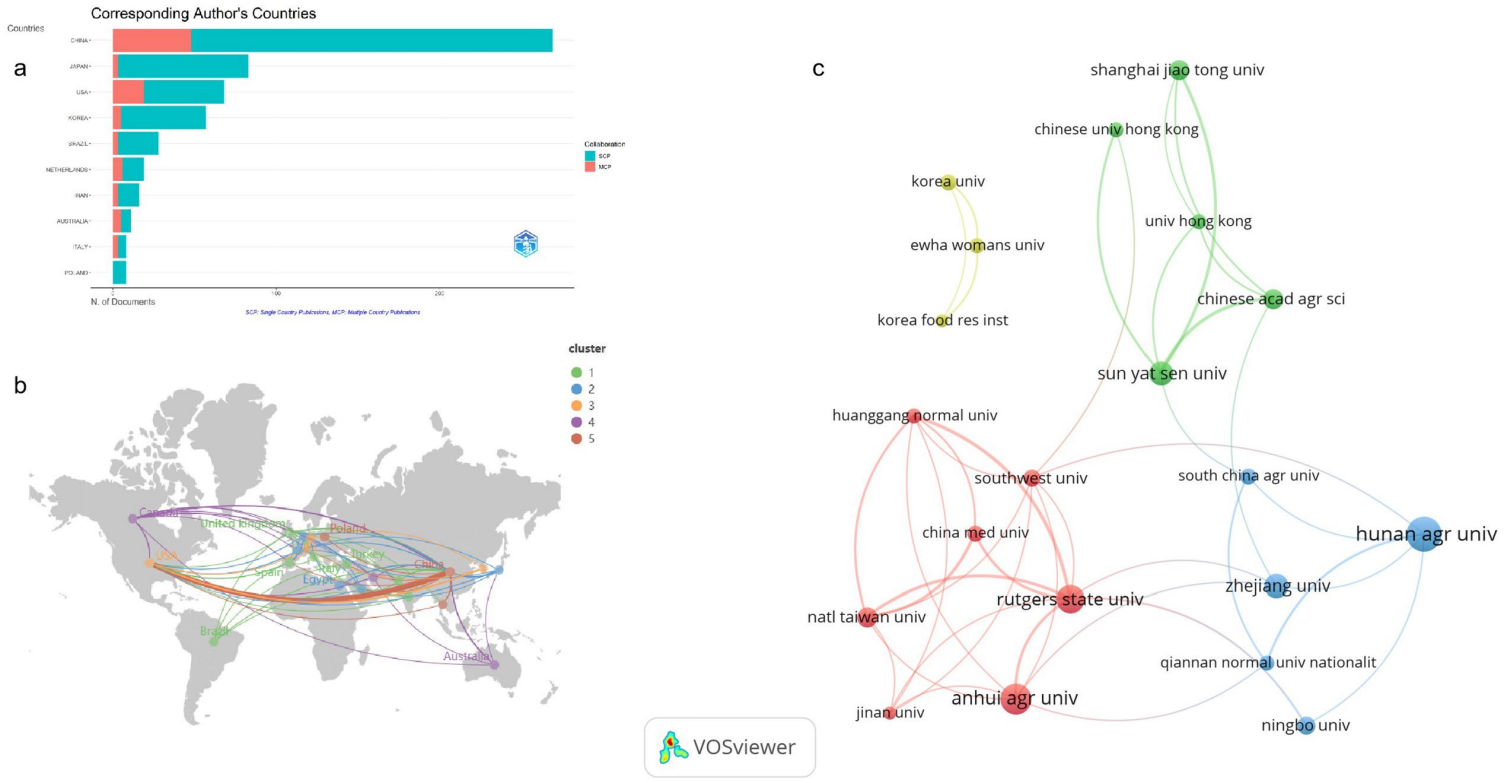
Analysis of countries and institutions publications. (a) Corresponding Author’s Countries. The figure’s blue segments illustrate SCPs, while the red segments represent MCPs. (b) Network diagram of collaborative relations between the top 22 most productive countries. The size of each node reflects the respective country’s publication count, with different colors distinguishing various clusters. The thickness of the lines between nodes indicates the level of collaborative intensity. (c) Collaboration of the top 20 most productive institutions.

Tea anti-obesity research spans 41 countries, displaying considerable variation in scholarly output. China, with 269 publications representing 40.7% of the total, leads in this research area, likely driven by its extensive tea culture (TC) and consumption. These publications have accrued 9,088 citations, averaging 33.8 per article, reflecting significant global recognition (Table 1). Japan and the United States are next, with 83 and 68 publications respectively, accounting for 12.6 and 10.3% of the total. The citation average for Japan is 51.5, whereas it is higher in the United States at 85.8, underscoring the significant impact of American research. South Korea ranks fourth with 57 contributions. Other nations such as Brazil, the Netherlands, and Iran have made smaller contributions, with 28, 19, and 16 publications, respectively. Notably, Switzerland, despite having only eight publications, boasts an average citation count of 205.1 per article, highlighting the notable attention its research has received in the academic sphere. Figure 3a depicts the distribution of tea anti-obesity research across these countries, differentiating between single-country publications (SCP) and multi-country collaborative publications (MCP). The top five countries generally have low MCP ratios; for instance, China’s MCP ratio is 17.84%, indicating a strong domestic focus but also significant international collaboration. Japan has the lowest MCP ratio at 3.61%, suggesting a reliance on national resources with minimal international engagement. Conversely, the United States, with more SCPs, shows a higher propensity for international collaboration with an MCP ratio of 27.94%. Figure 3b highlights the most robust collaboration between China and the U.S., as indicated by the thickest connecting line. Among the top 10 countries by publication volume, Australia and Italy display the highest MCP ratios at 45.45 and 37.5%, respectively, reflecting strong international cooperation in tea anti-obesity research.

**Table 1:** The top 10 most productive countries.

Rank	Country	Articles	Articles %	SCP	MCP	MCP %	TC	AC
1	China	269	40.70	221	48	17.84	9,088	33.8
2	Japan	83	12.56	80	3	3.61	4,276	51.5
3	USA	68	10.29	49	19	27.94	5,835	85.8
4	Korea	57	8.62	52	5	8.77	1,675	29.4
5	Brazil	28	4.24	25	3	10.71	739	26.4
6	Netherlands	19	2.87	13	6	31.58	2067	108.8
7	Iran	16	2.42	13	3	18.75	440	27.5
8	Australia	11	1.67	6	5	45.45	684	62.2
9	Italy	8	1.21	5	3	37.5	171	21.4
10	Poland	8	1.21	8	0	0	75	9.4

TC, total citations; AC, average citations.

A total of 899 institutions contributed to research in this field. Of the top 10 institutions, seven are located in China, accounting for 322 publications or 48.71% of the total (Supplementary Table S1). This underscores China’s pivotal role in tea-related anti-obesity research. Leading the count is Hunan Agricultural University with 96 publications, followed by Anhui Agricultural University with 75, and Yunnan Agricultural University with 33. The significant representation of agricultural universities highlights the importance of agrarian science in China, aligning with national priorities on agricultural science, technological innovation, and food security. Figure 3c delineates the collaborative networks of these institutions based on publication volume.

Within Cluster 1 (red), encompassing seven institutions, Rutgers State University is noted for its extensive collaborations within the cluster, as well as significant ties to Qiannan Normal University for Nationalities and Zhejiang University from other clusters, emphasizing its central role in the network. Cluster 2 (blue), consisting of five institutions, sees Qiannan Normal University for Nationalities demonstrating the closest cooperation with Hunan Agricultural University. In Cluster 3 (green), involving five institutions, Sun Yat-sen University forms key links with the Chinese Academy of Agricultural Sciences and Shanghai Jiao Tong University. Cluster 4 (yellow), which includes three institutions, features close collaboration among Ewha Womans University, Korea Food Research Institute, and Korea University.

### 3.3 Author and sources analysis

The quantity of publications and citations are crucial indicators of academic impact. In this domain, 3,215 authors have contributed, with Liu Zhonghua from Hunan Agricultural University leading with 15 publications, although these have accumulated a relatively modest 288 citations (Supplementary Table S2). Qitang He from Rutgers University holds second place with 13 publications, which have garnered 683 citations. Joshua Lambert from the State University of New Jersey, ranked fifth with nine publications, has a notable citation count of 1,009, reflecting the high esteem and practical relevance of his research.

**Figure 4:**
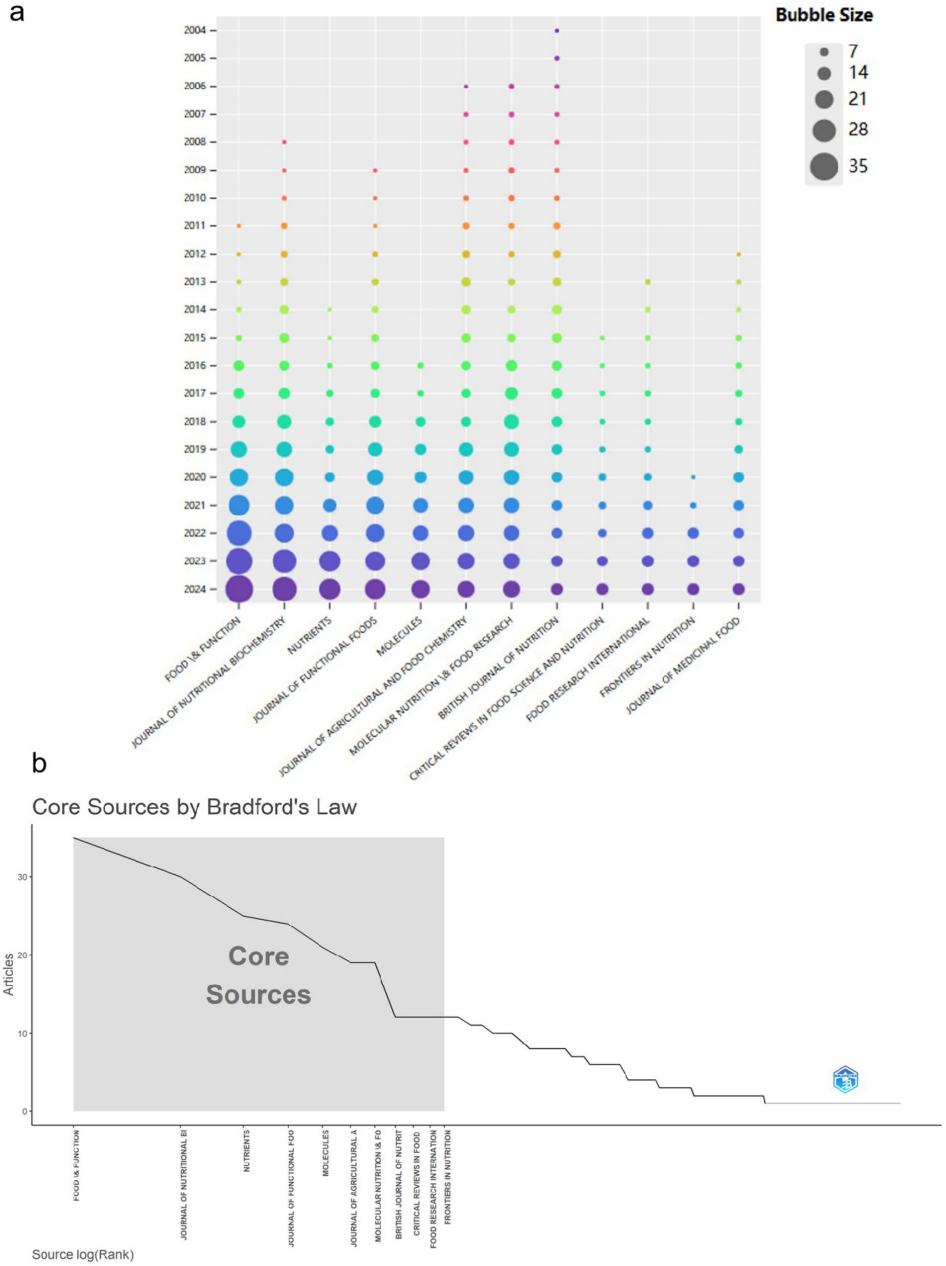
Analysis of sources publications. (a) Bubble plots depict the top 12 most productive sources. The legend categorizes the bubble sizes into five levels (7, 14, 21, 28, 35), with the bubble color gradually shifting from light to dark over time. (b) Core Sources according to Bradford’s Law.

In the realm of tea anti-obesity studies, the journal Food & Function is at the forefront with 35 pertinent publications (Supplementary Table S3). A high h-index (20) and g-index (33) indicate broad citation and recognition across the academic landscape. The Journal of Nutritional Biochemistry and Nutrients also play significant roles, with 30 and 25 publications respectively, emphasizing their impact in nutrition and functional foods research. As shown in Figure 4a, there is a consistent year-on-year increase in publication output across these journals. Bradford’s law, which describes the distribution of articles in scientific journals (75) (Figure 4b), where the gray areas represent core journals in the field.

### 3.4 Keywords analysis

**Figure 5:**
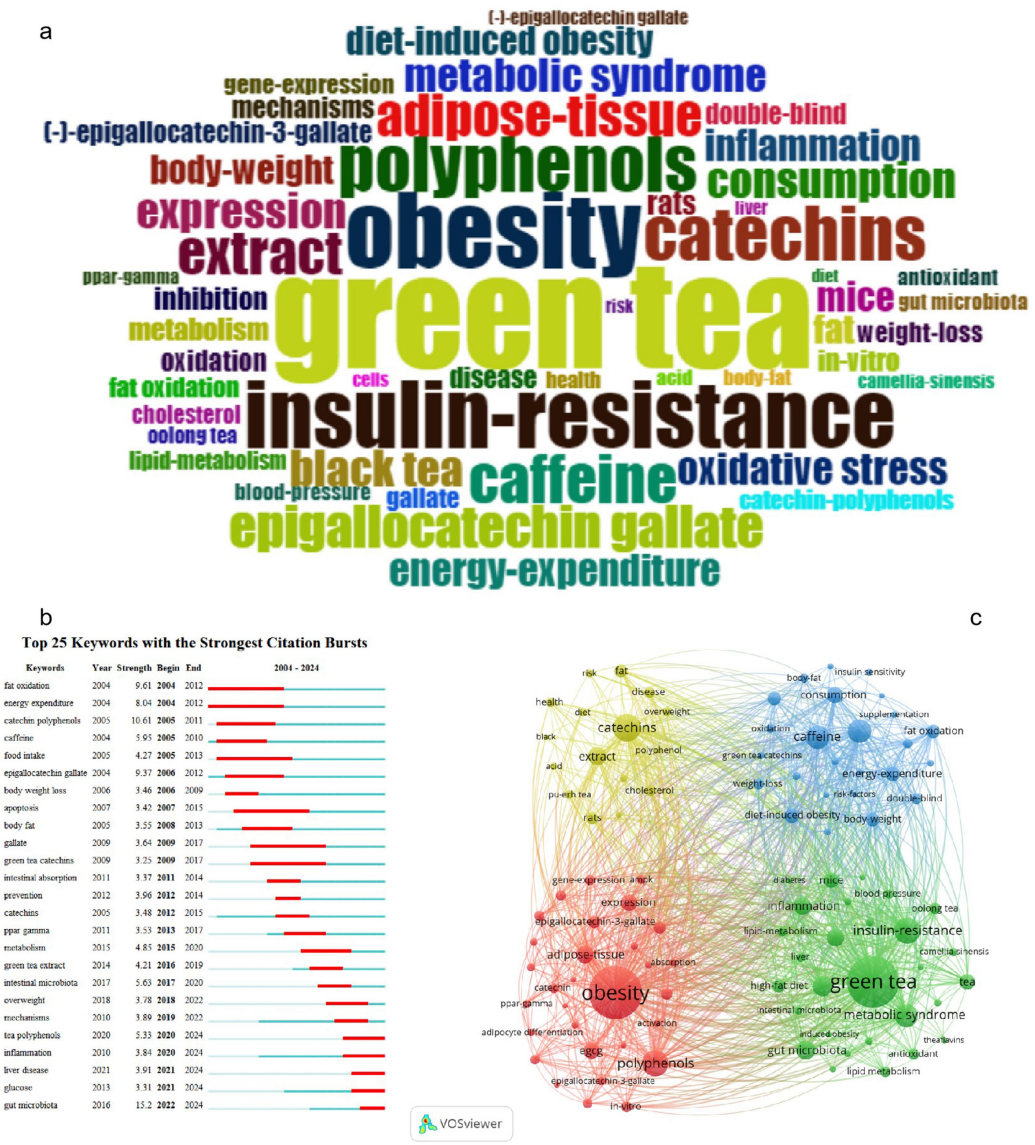
Keyword analysis. (a) Plus keyword cloud map. (b) Top 25 keywords with the strongest citation bursts. (c) Top 100 keyword co-occurrence map.

Keywords facilitate the identification and extraction of core themes and directions in research. By analyzing keyword frequency and co-occurrence, researchers can pinpoint emerging hotspots and trends. Keywords Plus, derived from titles, abstracts, and references, augments author-provided keywords to expand the scope of the search (76). The keyword plus cloud (Figure 5a) prominently features “green tea,” surrounded by related terms such as “obesity,” “metabolic syndrome,” “insulin resistance,” and “weight loss,” signifying a strong focus on the potential benefits of green tea for weight management and metabolic health. Additional keywords like “catechins,” “polyphenols,” and “epigallocatechin-3-gallate” (commonly abbreviated as EGCG), along with “oxidative stress,” “inflammation,” “lipid metabolism,” and “intestinal flora,” elucidate the mechanisms through which green tea components may exert anti-obesity effects.

Using Vosviewer we analyzed the top 10 keywords by occurrence frequency (Supplementary Table S4). “Obesity” emerged as the most frequent appearing 291 times underscoring its prominence in the literature. “Green tea” was a close second with 283 occurrences highlighting its vital role in anti-obesity research. “Black tea” ranked ninth with 80 mentions. Notably “catechins” were third with 121 occurrences while “EGCG” was seventh with 96. “Polyphenols” were fourth with 113 mentions “caffeine” ninth with 97 and “gut microbiota” tenth with 71. “Insulin resistance” and “metabolic syndrome” ranked fifth and eighth with 112 and 95 occurrences, respectively, indicating their significance in obesity-related research. This data suggests that tea components might help in preventing and treating obesity by improving insulin sensitivity.

A “citation burst” indicates a sharp increase in the citation frequency of specific keywords within a particular period, often signaling trending topics or emergent trends within a discipline. As illustrated in Figure 5b,” gut microbiota” topped the list with a burst intensity of 15.2 between 2022 and 2024, underscoring its increasing importance in obesity and metabolic health research. Research has established a substantial correlation between the composition of gut microbiota and the metabolic state of the host, implying its influence on obesity and associated conditions through the modulation of energy metabolism and inflammatory responses. “Catechin polyphenols” and “EGCG” were ranked second and fourth in citation intensity, respectively, highlighting the therapeutic potential of these tea constituents, renowned for their antioxidant, anti-inflammatory, and fat oxidation-enhancing properties. “Fat oxidation” and “energy expenditure” were ranked third and fifth, further accentuating the contribution of tea components to enhancing fat metabolism and energy expenditure.

Figure 5c presents an intricate keyword co-occurrence map of studies on tea’s anti-obesity effects, delineating four principal research areas (Supplementary Table S5). The first cluster (red, 27 clusters) is dedicated to metabolic regulation and cellular mechanisms, including keywords such as “obesity,” “epigallocatechin-3-gallate,” “adipogenesis,” “adipose tissue,” and “polyphenols.” The second cluster (blue, 25 clusters) pertains to population studies and weight management, encompassing “caffeine,” “energy expenditure,” “supplementation,” “consumption,” and “diet-induced obesity.” The third cluster (green, 24 clusters) centers on chronic diseases and metabolic health, featuring “green tea,” “metabolic syndrome,” “insulin resistance,” “inflammation,” and “gut microbiota.” The fourth cluster (yellow, 24 clusters) focuses on the antioxidant attributes of tea and its extensive impact on health, with keywords like “catechins,” “extract,” “polyphenol,” and “disease.”

### 3.5 Reference analysis

**Figure 6:**
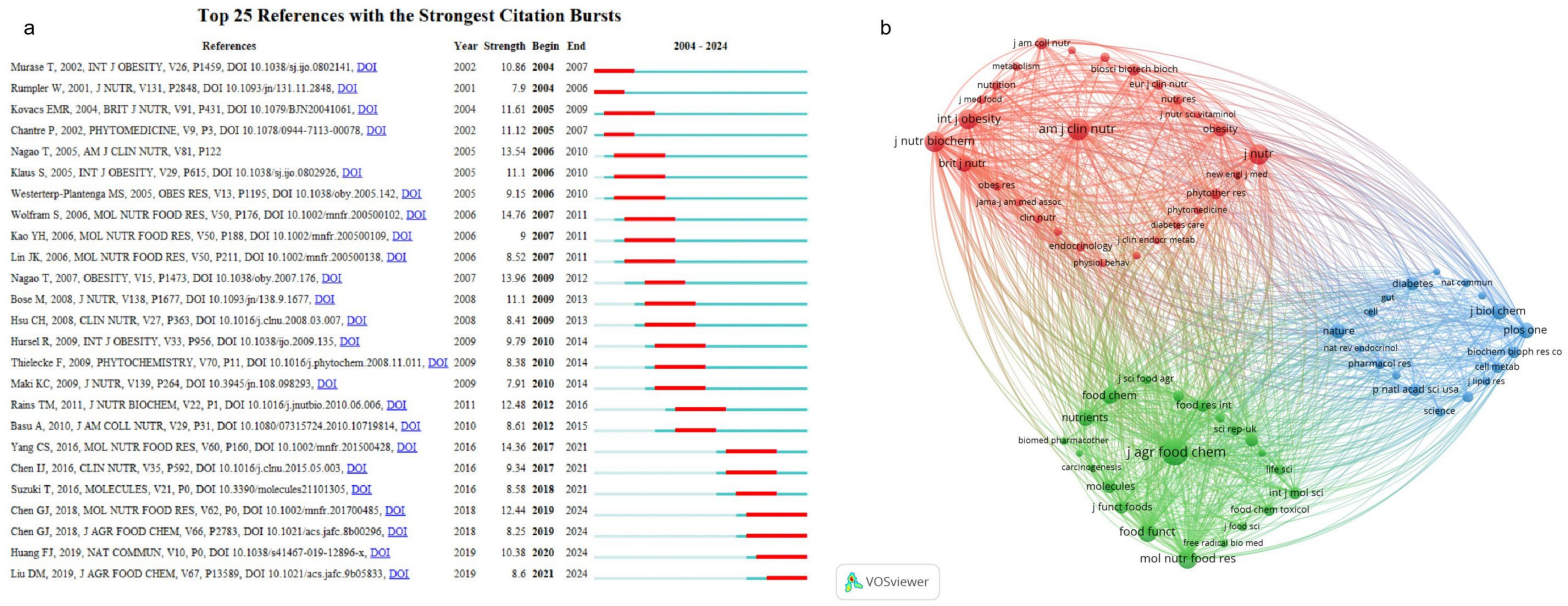
Reference analysis. (a) Co-occurrence of sources among the first 68 references. (b) Top 25 references with the strongest citation bursts.

The literature cited in tea anti-obesity research spans several disciplines, with a primary focus on clinical and nutritional sciences, food science and molecular nutrition, and biochemistry and molecular biology. This diversity highlights the research’s application in clinical settings and dietary interventions, as well as its exploration of molecular mechanisms and biochemical underpinnings. Journals such as the American Journal of Clinical Nutrition, Journal of Agricultural and Food Chemistry, and Nature exhibit high citation figures, reflecting the significant impact and broad academic attention garnered by this research, indicative of a trend toward multidisciplinary cross-research. The cited sources are divided into three groups (Figure 6a). The inaugural category (red, 28 entries) pertains to clinical and nutritional sciences. Esteemed publications include American Journal of Clinical Nutrition, American Journal of Physiology-Endocrinology and Metabolism, Annals of Nutrition and Metabolism, BioScience, Biotechnology and Biochemistry, British Journal of Nutrition, Clinical Nutrition, Diabetes Care, Endocrinology, European Journal of Clinical Nutrition Journal, International Journal of Obesity, Journal of the American College of Nutrition, Journal of Clinical Endocrinology and Metabolism, Journal of Medicinal Dietetics, Journal of Nutrition, Journal of Nutritional Biochemistry, Journal of Nutritional Sciences and Vitamins, JAMA, The Lancet, Metabolism, New England Journal of Medicine, Nutrition Research, Nutrition, Obesity Research, The Obesity Reviews, Obesity, Physiology and Behavior, Phytomedicine, and Phytotherapy Research. The American Journal of Clinical Nutrition and the Journal of Nutrition received 852 and 758 citations respectively, ranking them as the second and third most referenced journals (Supplementary Table S6), emphasizing the critical role of nutrition research in both clinical nutrition and public health sectors. The Journal of Nutritional Biochemistry stands as the fourth most-referenced journal, garnering 734 citations. The subsequent category (green, 22 references) is devoted to food science and molecular nutrition, including foremost journals such as Biomedicine and Pharmacotherapy, Carcinogenesis, Critical Reviews in Food Science and Nutrition, European Journal of Nutrition, European Journal of Pharmacology, Food Chemistry, Food and Chemical Toxicology, Food and Function, Food Research International, Free Radicals Biology and Medicine, International Journal of Biomacromolecules, International Journal of Molecular Sciences, Journal of Agricultural and Food Chemistry, Journal of Ethnopharmacology, Journal of Food Science, Journal of Functional Foods, Journal of Food and Agricultural Sciences, Life Sciences, Molecular Nutrition and Food Research, Molecules, Nutrients, Scientific Reports. The Journal of Agricultural and Food Chemistry is notable for the highest citation count (1,353) and total link strength (101,091), its significant influence on academic discourse. Molecular Nutrition and Food Research ranked fifth with 715 citations. The third category (blue, 18 items) delves into biochemistry and molecular biology, with prominent journals such as Biochemical and Biophysical Research Letters, Biochemical Pharmacology, Cell, Cell Metabolism, Diabetes Mellitus, The FASEB Journal, Gut, The Journal of Biological Chemistry, The Journal of Clinical Investigation, The Journal of Lipid Research, Nature Communications, Nature Medicine, The Nature Reviews Endocrinology, Nature, Proceedings of the National Academy of Sciences, Pharmacological Research, PLOS ONE, and Science.

The term “References with the Strongest Citation Bursts” denotes those publications experiencing a significant, sudden increase in citations within a specified timeframe, highlighting their pivotal role in sparking extensive interest and discussions across the academic landscape (77). The foremost citation burst (Figure 6b) transpired in the 2006 study by Wolfram et al. entitled “Anti-obesity effects of green tea: From bedside to bench” (78), reaching its peak between 2007 and 2011 with a burst score of 14.76. This review investigated how active tea compounds modulate fat metabolism, laying foundational insights for future inquiries.

Subsequently, Yang et al. analysis “Mechanisms of body weight reduction and metabolic syndrome alleviation by tea” (79) had a citation burst of 14.76 between 2017 and 2021. This article enhances our understanding of tea’s anti-obesity effects by delineating two main mechanisms: (i) tea components curtail the gut absorption of lipids and proteins, thus lowering calorie intake; (ii) TPs inhibit gluconeogenesis and fatty acid synthesis, boosting catabolism which ultimately aids in weight loss and remission of metabolic syndrome. The impact of these mechanisms may vary based on the type of tea ingested and individual dietary practices. Notably, Nagao’s 2007 and 2005 investigations, titled “A Green Tea Extract High in Catechins Reduces Body Fat and Cardiovascular Risks in Humans” (80) and “Ingestion of a Tea Rich in catechins leads to a reduction in body fat and malondialdehyde-modified LDL in men” (81), respectively, with citation bursts of 13.96 (2009–2012) and 13.54 (2006–2010), are significant. These clinical studies have shown that consistent consumption of catechin-rich green tea diminishes body fat, systolic blood pressure (SBP), and low-density lipoprotein cholesterol (LDL-C), underscoring its potential to mitigate obesity as well as cardiovascular disease risks.

The 2018 study by Chen et al., “Kudingcha and Fuzhuan Brick Tea Prevent Obesity and Modulate Gut Microbiota in High-Fat Diet Fed Mice” (82), ranked seventh in citation yet remains topical from 2019 to 2024, highlighting its continued relevance. This research investigates the effects of Kudingcha (KDC) and Fuzhuan Brick Tea (FBT) on the regulation of gut microbiota and obesity management. Results indicate that KDC decreases the relative abundance of the Danitaceae family, while FBT reduces the Firmicutes to Bacteroidetes ratio and enhances the relative abundance of Bifidobacterium, suggesting their role in mitigating metabolic syndrome features in mice on a HFD-fat diet by modulating the gut microbiota.

## 4 Discussion

### 4.1 Research overview and characteristics of publications

This study utilizes visualization and bibliometric analysis to outline the current research landscape concerning tea’s role in combating obesity. We document the yearly count of publications, along with data on contributing countries, institutions, journals, authors, references, and keywords, which elucidates the system, identifies research hotspots, and tracks the developmental trajectory of tea’s function against obesity, thus emphasizing the focal areas of this field. Drawing from the SCI-E and SSCI databases of WoSCC, we have compiled 661 papers and reviews from January 1, 2004, to June 30, 2024 (Figure 1). The persistent emphasis in this area is evidenced by a fluctuating yet growing trend in annual publications, with notable acceleration post-2016, marking the field’s ascension as a research hotspot.

A polynomial equation was employed to model the growth trajectory of the accumulated article count, achieving a high coefficient of determination (R^2^ = 0.9987) (Figure 2), which indicates robust predictive accuracy for future trends. This trend is propelled by: (1) the evolving recognition of TPs potential effects on obesity; (2) the intensifying global obesity crisis spurring the search for efficacious anti-obesity solutions; and (3) the inclination toward tea as a natural, side-effect-minimal option in line with current health trends. Although the annual growth rate of 5.86% signals active research, the steady development rate suggests the field remains exploratory, awaiting breakthroughs for accelerated progress.

The analysis (Figure 3) shows that China leads in tea and obesity research, followed by Japan, the USA, and South Korea, with Switzerland recording the highest average citation count per country, highlighting the high esteem and referential value of its research outputs. The national collaboration network indicates robust cooperation between China and the USA, with seven of the top 10 most prolific institutions based in China, where agricultural universities and research institutes play pivotal roles. This reflects China’s strong commitment to agricultural science and food research, especially concerning public health and nutrition. The institutional collaboration networks underscore the crucial role of international cooperation in driving this research area forward. Rutgers State University in the USA is particularly instrumental in fostering transnational research collaborations, facilitating knowledge exchange and advancing research.

Moreover, various Chinese institutions actively collaborate with global research entities, further bolstering China’s influence in the international tea anti-obesity research arena. Overall, the increasing globalization of the research network, with China at the helm, significantly contributes to the field’s rapid development. This global cooperation trend is poised to strengthen, underpinning more potential health applications of tea.

A total of 3,215 authors have engaged with the field of tea in combating obesity, with their influence measured by publication and citation counts. While Zhong Hualiu leads in publications, his relatively modest citation tally suggests limited research impact; conversely, Joshua Lambert’s substantial citations, despite fewer publications, denote his research’s high recognition and practical value.

An analysis of the distributed publications reveals 661 articles across 208 journals. Food and Function [IF(2023): 5.1, JCR: Q1] tops the list of journals in this research domain. Four of the top 10 journals boast impact factors above 5, with two exceeding 7 (Supplementary Table S3), indicative of the area’s propensity for more profound or innovative research. Predominantly, journals within the Q1 quartile host these high-impact studies, reflecting their broad acknowledgment and citation within the research community.

### 4.2 Research current status and trends

Reference co-citation and high-frequency keyword analyses have been utilized to delineate the principal areas of research within the domain of tea and obesity. Over the last two decades, “gut microbiota” has emerged as the keyword with the most significant intensity, peaking at 15.2 between 2022 and 2024; “catechin polyphenols” and “EGCG” ranked second and fourth, respectively, while “fat oxidation” and “energy expenditure” ranked third and fifth (Figure 5b). The research primarily focuses on the mechanisms through which tea constituents modulate intestinal microbiota, promote fat oxidation, and enhance energy expenditure to exert anti-obesity effects.

The timeline of Strongest Citation Bursts reveals evolving trends within the field. An initial review by Wolfram et al. (Figure 6b) in 2006 explored the impact of active green tea components, especially catechins, on fat metabolism, laying the groundwork for future studies on green tea’s anti-obesity properties. Subsequent clinical research by Nagao et al. from 2005 to 2007 substantiated the efficacy of green tea in reducing body fat and cardiovascular risks. Yang et al.’s study in 2016 further elucidated tea’s mechanisms, particularly its role in decreasing lipid absorption and activating AMPK. More recently, a 2018 study by Chen et al. has been instrumental in assessing tea’s capacity to counter obesity by influencing gut microbiota, with Citation Bursts spanning from 2019 to 2024, underscoring the persistent focus on gut microbiota in tea anti-obesity research.

The trajectory of tea anti-obesity research has progressively evolved from initial basic mechanism exploration and clinical studies to more in-depth mechanistic investigations in recent years. The modulation of gut microbiota has emerged as a focal point of research, with upcoming investigations expected to explore the interactions between tea constituents and gut microbiota in order to develop innovative anti-obesity approaches or functional food products.

#### 4.2.1 Main anti-obesity components in tea

Tea, recognized as a natural and beneficial beverage, primarily attributes its anti-obesity effects to key components such as TPs, caffeine, amino acids (like L-theanine), thearubigins, and tea polysaccharides. These constituents collaboratively function through diverse mechanisms to regulate body weight and ameliorate health issues linked to obesity, with TPs and caffeine identified as the most potent agents for weight reduction (83). TPs, one of the main anti-obesity agents, benefit bone, liver, kidney, cardiovascular health, gut microbiota, and sleep quality (84). These include catechins, theaflavins, tannins, and flavonoids (85, 86), originate from various phenolics in tea and are present in multiple forms in both green and black teas. For instance, green tea comprises approximately 30–40% of its dry weight as catechins, which can convert into theaflavins during the fermentation process, predominantly found in black tea (87). Catechins’ anti-obesity properties are partly attributed to their beneficial effects on the gut microbiome, reduction in hepatic steatosis, and anti-inflammatory actions (88). EGCG is particularly noted for its significant role in the prevention and treatment of obesity (54). Theaflavins contribute to weight management by reducing food intake, inhibiting pancreatic lipase activity, activating AMPK proteins, and regulating gut microbiota (89). Research indicates that theaflavins TF1, TF2a, and TF3 enhance glucose and lipid metabolism in mice on a HFD by activating the SIRT6/AMPK/SREBP-1/FASN pathway, thus effectively mitigating obesity symptoms (90).

Caffeine, a natural plant-derived active compound known chemically as 1,3,7-trimethylxanthine, addresses obesity by affecting fat cell differentiation, boosting thermogenesis and fat breakdown, suppressing appetite, and reducing inflammation (91). Wu et al. (92) reported that caffeine diminishes appetite; Liu et al. (93) observed that it may decrease inflammation levels and suppress the expression of genes associated with fat production (such as SREBP1c, FAS, and ACC) and inflammatory markers (TNFα, MCP-1, and IL-6). L-theanine, an amino acid found naturally in tea leaves (94), has demonstrated substantial potential in regulating lipid metabolism. It influences gut microbiota and bile acid (BA) metabolism through the FXR-FGF15-CYP7A1 pathway (95). Tea pigments, which impart specific hues to tea, offer health benefits including anti-obesity, anti-tumor, anti-inflammatory, antiviral, antioxidant, and antibacterial properties. Research has shown that raw Pu-erh tea brown pigment (R-TB) more effectively regulates blood sugar, reduces inflammation, and inhibits genes and proteins associated with fat production, whereas fermented Pu-erh tea brown pigment (F-TB) more effectively curtails genes and proteins linked to fat breakdown (96).

Tea polysaccharides (TPSs), significant bioactive components in tea, have been extensively researched for their antioxidant, anticancer, blood sugar-lowering, anti-fatigue, anticoagulant, anti-obesity, and immunomodulatory properties (97). Research demonstrated that a novel set of Fu brick tea polysaccharides (FTPS) extracted from Fuquan brick tea markedly reduced lipid levels in HepG2 cells treated with oleic acid compared to those treated with FTP3 (98). Hence, TPs, caffeine, L-theanine, and tea polysaccharides are the primary contributors to tea’s anti-obesity effects. Researchers also associate the anti-obesity properties of black tea with its alkaloid content (99). Indigestible substances in tea, such as cellulose, serve as prebiotics, further enhancing health by modulating gut microbiota and other mechanisms. These components display considerable potential and broad prospects for application in obesity research.

#### 4.2.2 Regulation of gut microbiota

The human gut harbors a vast ecosystem, comprising billions of microorganisms including bacteria, fungi, and viruses, collectively known as gut microbiota (100). These microorganisms are crucial for digesting nutrients, metabolizing energy, glucose, and cholesterol, and modulating chronic inflammation, significantly affecting the interplay between diet, obesity, and related diseases (101, 102). Research indicates that obese individuals typically have higher levels of bacteria like Lactobacillus, *Escherichia coli*, and Bacteroides, which are more efficient at energy harvesting, whereas levels of Bifidobacterium, which enhances gut function, are lower (103). Studies involving mice on HFDs have shown marked gut microbiota dysbiosis, notably an increase in the Firmicutes/Bacteroidetes ratio (F/B ratio) (104). Reducing this ratio may aid obesity treatment, as supported by various studies (105). Clinical trials by Ley et al. (106) observed higher proportions of Firmicutes in obese subjects, which decreased following weight loss, thereby increasing Bacteroidetes levels. However, further research and meta-analyses have yet to confirm a definitive link between the F/B ratio and obesity (107), indicating that the relationship remains debatable and necessitates further exploration (108).

Tea has been found to enhance beneficial bacteria associated with favorable obesity outcomes, such as Alistipes and Lachnospiraceae, and suppress those positively correlated with obesity, thereby improving the gut microbial community structure (105). Additionally, metabolic byproducts of the gut microbiota, namely Short Chain Fatty Acids (SCFAs) including acetates, propionates, and butyrates, not only stimulate thermogenesis and fatty acid oxidation to reduce fat formation but also promote leptin secretion via G-protein coupled receptor 43 (GPR43), enhancing satiety and reducing food intake (109). SCFAs also elevate glucagon-like peptide-1 (GLP-1) and peptide YY (PYY) levels in the blood through G-protein coupled receptor 41 (GPR41), which enhances gut motility and appetite control via the arcuate nucleus of the hypothalamus (110). Research by Liao et al. (111) confirmed that green tea increases Bifidobacteria levels in the gut microbiota of mice, which produce SCFAs. Additional studies have shown that combining brick tea with gut microbiota reconstruction encourages fat browning and thermogenesis, offering potential strategies for addressing obesity and its associated metabolic diseases (112).

The principal agents in tea promoting weight loss through gut microbiota regulation are TPs and tea polysaccharides. Other ingredients, such as tea pigments, caffeine, and theanine, also positively influence gut health, although research on these components is still relatively limited.

##### 4.2.2.1 TPs regulate gut microbiota

Extensive studies have shown that TPs can modulate obesity by promoting or inhibiting the growth of specific gut bacteria (113). TPs directly influence the gut redox state and microbial community abundance (114), and are also transformed by the gut microbiota, affecting lipid metabolism processes. Research comparing the effects of TPs on conventional mice (CVZ) and pseudo-germ-free mice (PGF, treated with antibiotics) in terms of weight loss and lipid metabolism showed that TPs had a less pronounced anti-obesity effect on PGF-obese mice compared to obese CVZ mice, underscoring the significant role of gut microbiota in the impact of TPs on obesity, and suggesting that their anti-obesity effects might be diminished without a typical microbial community (115). It is believed that most TPs, possibly over 80%, remain in the gut where they are metabolized into short-chain fatty acids and phenolic acids by esterases and glucosidases produced by the gut microbiota (116), thereby enhancing the body’s capacity to break down and utilize these polyphenols, increasing the proportion of active substances involved in metabolic regulation (116). Many experiments have proven the regulatory effects of TPs on gut microbiota.

Research by Liu et al. (117) on the *in vitro* interactions between EGCG and human gut microbiota demonstrated that EGCG treatment stimulates beneficial bacteria such as Christensenellaceae, Bifidobacterium, and Bacteroides, and inhibit pathogens like Bilophila, Enterobacteriaceae, and *Fusobacterium* var*ium*. Bacteroides and Bifidobacterium increase the production of secondary bile acids in the gut, impacting host metabolism in several crucial ways and playing an essential role in regulating lipid and glucose metabolism and energy balance.

Experiments by Li et al. (118) showed that TPs significantly mitigate the reduction in gut microbiota abundance and diversity caused by antibiotics, and increase the relative abundance of probiotics such as Eubacterium, Roseburia, and Lactobacillus. Gut microbiota, including Lactobacilli and Bifidobacteria, are primary sources of short-chain fatty acids (119). Studies have proven that TP significantly boost the number of beneficial gut bacteria Akkermansia, which regulates host lipid balance through interactions with lipid metabolites, reducing endotoxin levels, increasing short-chain fatty acid production, and promoting fatty acid oxidation (120, 121). Additionally, Hussain et al. (115) found that compared with germ-free mice, the ratio of Firmicutes to Bacteroidetes in conventional mice significantly decreased after catechin intake, and there was a significant reduction in epididymal fat, liver weight, glucose levels, total cholesterol, and high-density lipoprotein cholesterol levels. Thus, beyond directly regulating gut microbiota structure, TPs influence obesity-related diseases by affecting the production of gut microbial metabolites, including SCFAs and secondary bile acids. Furthermore, TPs can reduce weight by enhancing gut permeability, lowering endotoxin circulation levels, and alleviating inflammatory responses (122). Noel et al. (123) discovered that TPs alter the tricarboxylic acid (TCA) cycle and urea cycle of gut microbiota in rats, thereby enhancing the energy conversion efficiency of rats, which aids in reducing blood sugar and cholesterol levels.

##### 4.2.2.2 Tea polysaccharides regulate gut microbiota

As tea polysaccharides pass through the gastrointestinal tract, they undergo microbial breakdown into smaller sugar units. These microbes then utilize these sugars and produce beneficial short-chain fatty acids (SCFAs) (37, 124). Consequently, tea polysaccharides primarily influence gut microbiota by serving as prebiotics, which facilitates the production of bacterial metabolic products such as SCFAs—acetate, propionate, and butyrate. These changes in microbial activity subsequently alter the composition of the gut microbiota (125), leading to improvements in obesity management (126). Furthermore, studies have shown that the *in vitro* fermentation products of tea polysaccharides exhibit anti-inflammatory effects on LPS-treated RAW264.7 macrophages, likely due to the SCFAs present in these products (127). In models of dextran sulfate sodium-induced colitis (128), tea polysaccharides have also been found to promote the growth of Bacteroides genus, which reduces levels of LPS in feces and plasma, thereby enhancing the intestinal epithelial barrier function and reducing both intestinal and systemic inflammation.

Taiping Houkui tea polysaccharides have shown potential in influencing lipid metabolism by reshaping the gut microbiota and its metabolic byproducts (129). In animal models, tea polysaccharides have effectively counteracted disruptions in gut microbiota in hyperlipidemic rats, reduced the Firmicutes to Bacteroidetes ratio, and increased the relative abundance of efficient SCFA producers such as *Lachnospira* sp., *Victivallis*, and *Rossella* spp. These microbiota alterations contribute to reducing cholesterol accumulation and fat synthesis in rats, thus aiding in the regulation of blood lipid levels (130). Research indicates that Ziyang selenium-rich green tea polysaccharides (Se-GTP) significantly raise the levels of succinate—a microbial metabolite linked to the thermogenesis of fat cells—in the colon of obese mice (131). Fu brick tea polysaccharides have been noted to promote adipocyte browning and thermogenesis by modulating gut microbiota, thereby offering a preventative measure against obesity (132). The anti-obesity impact of polysaccharides in dark tea (hmtp) correlates strongly with shifts in the relative abundance of gut microbiota, particularly with marked increases in Dubosiella and Romboutsia, which inversely correlate with body weight and positively with the IBAT index. Such microbial changes may trigger browning of inguinal white adipose tissue (iwat) and boost the thermogenic activity of brown adipose tissue (ibat) by upregulating thermogenic genes like UCP1, PRDM16, and PGC1 alpha (133). Additionally, TPs may decrease body weight by improving gut permeability, reducing endotoxin circulation levels, and alleviating inflammatory responses (122).

##### 4.2.2.3 Other active ingredients regulate gut microbiota

Tea pigments, such as theaflavins, thearubigins, and theabrownins, regulate the abundance, diversity, and structure of gut microbiota, thereby aiding in weight loss (134). Theabrownin extract enhances the presence of beneficial gut bacteria like Prevotella and Bacteroides in individuals with metabolic diseases, boosts the production of SCFAs, aids in the breakdown of proteins and carbohydrates, improves insulin resistance, and significantly lowers blood sugar and lipid levels (135). In addition, theabrownins influence obesity and related diseases by modulating bile acid metabolism mediated by gut microbiota. The transformation of cholesterol into bile acids in the liver, via the classical pathway that produces 12α-hydroxy bile acids (cholic acid) catalyzed by CYP8B1 and the alternative pathway that yields non-12α-hydroxy bile acids (chenodeoxycholic acid) catalyzed by CYP7B1, constitutes the primary cholesterol metabolism route (136). Research has also shown that theabrownins elevate the abundance of Desulfovibrio and Clostridium, which are involved in the synthesis of secondary bile acids that activate the intestinal FXR signaling pathway by enhancing 7α-dehydroxylation, thus ameliorating lipid metabolic disorders (137, 138).

Further study reveal that a Fubrick tea supplement mitigated obesity induced by a HFD by altering gut microbiota and increased serum levels of caffeine, theophylline, and theobromine, correlating positively with the abundance of the Lachnospiraceae bacterial group (139). Post-intervention with high-caffeine tea, mice on a HFD exhibited a significant rise in Prevotella, a butyrate-producing bacterium known for its anti-inflammatory effects and enhanced energy metabolism (140). L-theanine has demonstrated efficacy in boosting the relative abundance of obesity-related probiotics such as Oscillibacter and Lactobacillus, while suppressing pathogens, thus mitigating obesity-induced gut microbiota dysbiosis (94).

#### 4.2.3 Anti-obesity mechanisms

Tea and its components play a multifaceted role in obesity management through mechanisms such as modulating lipid metabolism, controlling dietary intake, adjusting energy metabolism, and managing oxidative stress and inflammation (89, 141).

##### 4.2.3.1 Limiting dietary intake

Increasing food intake significantly contributes to obesity (142). Conversely, managing food intake is vital for preventing weight gain and involves regulating food consumption, selection, and absorption. Food intake correlates strongly with insulin sensitivity, leptin activity, and hypothalamic regulation (143, 144). Insulin regulates glucose homeostasis and fat metabolism through the hypothalamus, also influencing food intake. Leptin boosts lipid metabolism, inhibits fat formation, and supports related hypothalamic functions (145).

Studies demonstrate that tea and its components impact neuroendocrine metabolic regulators of appetite, reducing food consumption and limiting the gastrointestinal absorption of nutrients (146, 147). Theaflavins enhance insulin sensitivity by promoting protein kinase B signaling, reducing glucose toxicity, and suppressing inflammation (89). Matcha green tea is crucial in preventing obesity-induced hypothalamic inflammation by blocking the JAK2/STAT3 signaling pathway, thereby aiding in the management of obesity-related metabolic syndrome. This inhibition of inflammation-related pathways can restore normal hypothalamic functions, enhancing overall metabolic health (148). Feeding mice a 4% green tea diet for 16 weeks significantly reduced both food intake and serum leptin levels, leading to lower serum lipid levels (149). Additionally, intraperitoneal injection of green tea catechins reduced food intake by 50 to 60% in both lean and obese rats (150). Caffeine may suppress appetite by impacting hormones such as ghrelin and leptin. Combined administration of EGCG and caffeine not only prevents fat accumulation but also promotes anorexic effects in mice, elevating GLP-1 and POMC levels in the hypothalamus (151). However, the influence of green tea on reducing fat is noted to be independent of energy intake, suggesting a minimal impact on food consumption (152, 153). The effect of green tea on satiety is ambiguous and may vary based on dosage and method of administration (149, 150). Therefore, further investigations are necessary to understand the influence of factors such as gender, age, and dosage on green tea’s properties.

##### 4.2.3.2 Inhibition of lipid absorption and synthesis

Lipid accumulation, a marker of overweight and obesity, results from increased fat synthesis, fatty acid intake, or diminished fatty acid oxidation (154). Inhibiting fat absorption and synthesis directly reduces body fat accumulation. Numerous studies confirm the efficacy of tea and its components in decreasing human fat storage and body weight (21, 79, 155). Tea components reduce the emulsification and absorption of lipids and proteins within the gastrointestinal tract, thereby lowering caloric intake (146, 156). They inhibit the differentiation and proliferation of preadipocytes (156), reducing lipid production (79, 147). Drinking Citrus Pu-erh Tea (CPT) before or after meals effectively lessens fat digestion in the small intestine, alleviating obesity due to excessive fat absorption (157). Consuming a blend of catechin-rich green tea extract and by-products like flavonols and polysaccharides blocks lipid absorption in the intestines, preventing accumulation in fat cells (158).

Another pathway to reduce lipid synthesis involves downregulating fatty acids. Research has demonstrated that tea brownie leads to a downregulation of proteins and mRNA expressions involved in fatty acid synthesis and lipid production (159). Both native and thermally modified catechins (TMC) are capable of downregulating endogenous fatty acid synthesis (160). Ganpu tea (Mandarin Pu-erh Tea) significantly reduces inflammatory cytokine levels induced by a HFD, diminishing lipid droplets and curtailing the expansion of white adipose tissue (161). Furthermore, tea affects the differentiation, growth, and deposition of fat cells. It has been shown that EGCG inhibits the differentiation of adipose-derived mesenchymal stem cells into fat cells (162). Additionally, it restricts the growth of 3 T3-L1 cells through the miR-143/MAPK7 pathway (163). A combination of tea leaves and citrus (grapefruit) reduces lipid deposition in HepG2 cells via the AMPK/ACC pathway (164). Research also indicates that Liu Bao tea water extract (LTWE) prevents the proliferation and differentiation of preadipocytes by regulating the gene expression of transcription factors involved in fat generation and pro-inflammatory factors (165).

##### 4.2.3.3 Promote the metabolism of energy substances

Boosting fat consumption is essential for weight loss as it helps reduce body fat and body weight, improves metabolic health, and lowers disease risk. Additionally, increasing fat consumption can enhance basal metabolic rate, improve exercise performance, and refine body composition. Research shows that tea and its components stimulate fat breakdown, lipid metabolism (79, 156), and fecal lipid excretion (146). Gamma-aminobutyric acid (GABA), extracted from oolong tea, promotes thermogenic proteins like uncoupling protein-1 and peroxisome proliferator-activated receptor-*γ* coactivator (PGC-1α), enhancing lipid metabolism and fatty acid oxidation (166).

Enhancing the number and function of mitochondria is closely linked to fat breakdown. Mitochondria promote fat oxidation, improve fat-burning efficiency, reduce body fat storage, and support energy production and metabolic health, maintaining the normal function of fat cells and overall metabolic balance. Treatment with heat-treated green tea extract (HTGT) and enzymatically modified isoquercitrin (EMIQ) is more effective than orlistat in combating obesity. In treated groups, genes related to mitochondrial oxidative metabolism and PKA signaling, associated with fat breakdown, showed dose-dependent upregulation, suggesting that this treatment enhances energy metabolism and glucose tolerance by boosting mitochondrial function and fat breakdown (167). Additional research indicates that HTGT and EMIQ have *in vivo* anti-obesity effects, partly by increasing mitochondrial metabolism in fat cells (168). Furthermore, studies have shown that EGCG mitigates palmitate-induced muscle atrophy by regulating mitochondrial function in C2C12 cells, increasing energy expenditure and mitochondrial content in mice to prevent obesity (169).

Brown fat cells generate heat by metabolizing fat, thereby increasing energy expenditure and reducing fat accumulation (170). Consequently, increasing the activity and number of brown fat cells is a key focus in contemporary obesity management research. Studies have shown that tea and its components facilitate the transformation of white adipose tissue to brown, enhancing its oxidation, combustion, and energy consumption through heat generation (146, 171). EGCG plays a role in anti-obesity by modulating the activity of both white and brown adipose tissues (172). Evidence suggests that EGCG significantly promotes anti-obesity effects by upregulating Beclin1-dependent autophagy and lipid breakdown in white adipose tissue (173). Additionally, studies have found that selenium-enriched green tea polysaccharides (SE-GTP) stimulate thermogenesis in brown adipose tissue (BAT) and the browning of inguinal white adipose tissue (IWAT) in obese mice by boosting the expression of thermogenesis-related marker proteins UCP1, PGC-1*α*, and Cidea in BAT and IWAT (131). Fu brick tea extract also increases energy expenditure and promotes the browning of subcutaneous adipose tissue by upregulating the expression of specific genes, such as uncoupling protein 1, effectively preventing weight gain (174).

##### 4.2.3.4 Regulation of oxidative stress and inflammatory Levels

Obesity is recognized as a state of chronic low-grade inflammation where adipocytes secrete pro-inflammatory cytokines such as TNF-α, IL-6, and IL-8 (175). Modulating oxidative stress and inflammation is critical for potential anti-obesity agents (79). Tea extracts exhibit strong anti-inflammatory and antioxidant properties, aiding in weight management and alleviating various chronic diseases (26). Matcha green tea inhibits the JAK2/STAT3 pathway, preventing hypothalamic inflammation induced by obesity (148). Aged green tea activates the AMP-activated protein kinase pathway, reducing fat accumulation and inflammatory responses prompted by a HFD (176). L-theanine effectively decreases inflammation and enhances metabolism by blocking the phosphorylation of key proteins in the NF-kappa B/mitogen-activated protein kinase (MAPK) pathway, thus preventing obesity in rats on a HFD (94). TP lower systemic LPS levels and curb the LPS-activated TLR4/NF-kappa B pathway, reducing inflammation linked to obesity (177). Tea components such as theanine and caffeine bolster antioxidant capabilities, amplify free radical scavenging activity, and lessen oxidative stress (178, 179).

The immune environment is crucial in obesity and metabolic syndrome, and modulating immune cells within adipose tissue can ameliorate metabolic issues. M2 macrophages foster anti-inflammatory responses and fat metabolism, enhancing insulin sensitivity. Polysaccharides from Da-Huang tea may aid in weight loss and improve metabolic syndrome by promoting the polarization of macrophages toward the M2 type (180). Further research shows that selenium-enriched green tea polysaccharides (SE-GTP) significantly increase the number of M2 macrophages in IWAT, which possess anti-inflammatory properties, promote metabolism, and enhance the thermogenic capacity of fatty tissue (131). Tibetan tea reduces chronic inflammation by suppressing inflammatory responses in white adipose tissue, decreasing adipocyte proliferation and immune cell infiltration. It also induces metabolic remodeling, promotes glutamine synthesis, and inhibits the production of pro-inflammatory chemokines, effectively aiding in weight loss (181).

## 5 Conclusion

This study utilized CiteSpace, VOSviewer, and bibliometrics online analysis platforms to thoroughly assess the current status, hotspots, and trends in tea anti-obesity research. Although China is leading in this domain, collaboration and communication among countries, institutions, and authors are still inadequate. While there has been progress in the research on preventing and treating obesity and its associated metabolic diseases, significant breakthroughs have yet to be achieved, indicating that the research remains largely exploratory. Further advancements in key areas are essential to hasten research progress, especially in deeper mechanistic studies and interdisciplinary collaboration. In conclusion, this bibliometric study offers a comprehensive overview of the field and provides valuable insights for future research.

## Supplementary material

Supplementary Figure S1 Main information for details.

Supplementary Table S1 Affiliations.

Supplementary Table S2 Top 10 most productive authors.

Supplementary Table S3 Top 12 most productive sources.

Supplementary Table S4 Top 10 most productive keywords.

Supplementary Table S5 The top 100 keyword clusters.

Supplementary Table S6 Co-occurring sources of top 68 references.
