# Supplementary figures and images for: Global hotspots and trends in tea anti-obesity research: a bibliometric analysis from 2004 to 2024
Source: Front Nutr. 2024 Nov 13;11:1496582. doi: 10.3389/fnut.2024.1496582 (PMC11598529; doi:10.3389/fnut.2024.1496582)

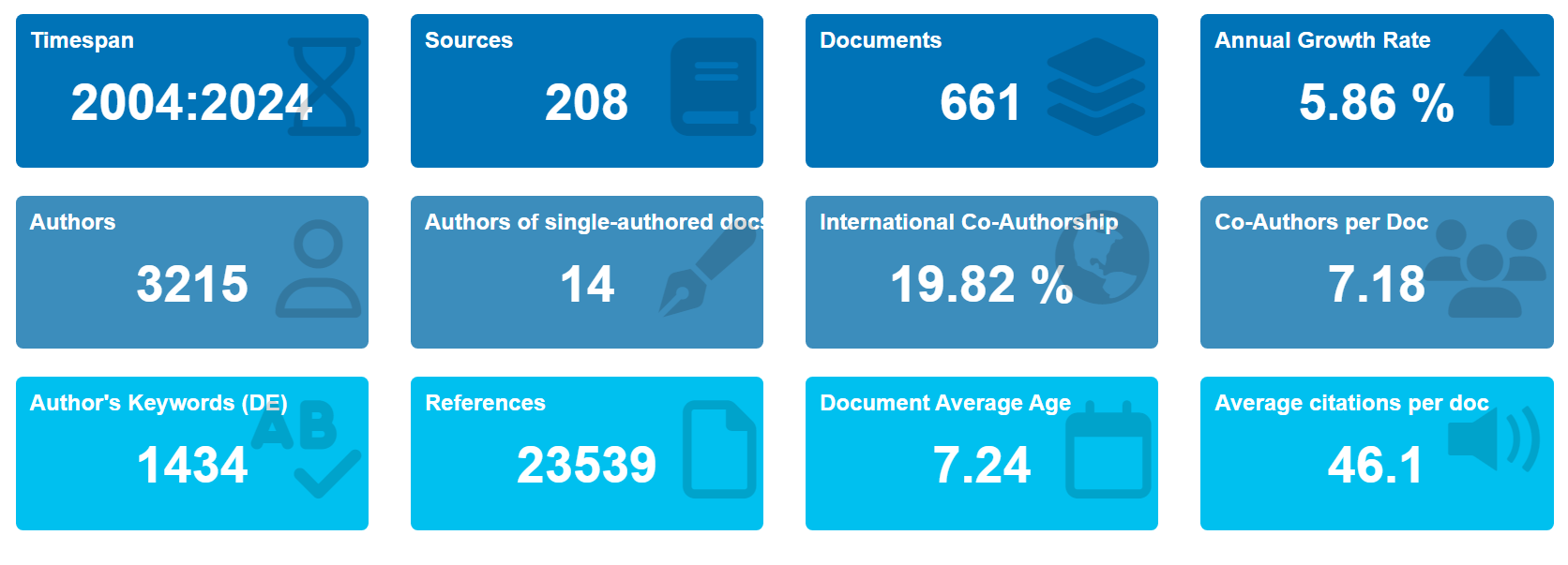

Supplement: Supplementary Figure S1 — Main information for details. [file Image_1.PNG]
